# Changes in Sedentary Time and Implicit Preference for Sedentary Behaviors in Response to a One-Month Educational Intervention in Primary School Children: Results from the Globe Trotter Pilot Cluster-Randomized Study

**DOI:** 10.3390/ijerph20021089

**Published:** 2023-01-07

**Authors:** Alicia Fillon, Nicole Fearnbach, Stéphanie Vieira, Jade Gélinier, Sarah Bagot, Mélina Bailly, Audrey Boscaro, Léna Pélissier, Julie Siroux, Vincent Grasteau, Jean Bertsch, Bruno Pereira, Martine Duclos, Céline Lambert, David Thivel

**Affiliations:** 1 Laboratory of the Metabolic Adaptations to Exercise under Physiological and Pathological Conditions (AME2P), EA 3533, Clermont Auvergne University, CRNH Auvergne, 63178 Clermont-Ferrand, France; 2 National Observatory for Physical Activity and Sedentary Behaviors (ONAPS), UFR Medicine, University Clermont Auvergne, 28 Place Henri Dunant, 63000 Clermont-Ferrand, France; 3 Office of Research, Florida State University, Tallahassee, FL 32306, USA; 4 Clinical Sciences Division, Pennington Biomedical Research Center, Baton Rouge, LA 70808, USA; 5 Fondation JDB Pour Prevention Cancer, 91640 Fontenay-Lès-Briis, France; 6 Biostatistics Unit (DRCI), Clermont-Ferrand University Hospital, 63000 Clermont-Ferrand, France; 7 Department of Sport Medicine and Functional Explorations, Clermont-Ferrand University Hospital, G. Montpied, 63000 Clermont-Ferrand, France

**Keywords:** physical activity, sedentary behaviors, primary school children, educational intervention, prevention

## Abstract

School-based multi-component educational interventions have been encouraged to improve children’s movement behaviors. The present study evaluates the effect of the Globe Trotter Initiative on physical activity (PA) level, sedentary time, physical fitness and activity preferences in primary school children. A total of 361 children (9–10 years) participated in this cluster-randomized trial. Nine schools were randomized as control (CON, 121 children) or Globe Trotter schools (GT, 240 children). Physical fitness, body composition, anthropometric characteristics, PA level, sedentary behaviors, physical self-perception, and activity preferences were evaluated at baseline (T0) and after the one-month intervention (T1). Grip strength performance and overall completion time of the obstacle course show a significant time effect (*p* < 0.001) in both groups (no group effect). PA level and physical self-perception did not significantly show time nor group effects. The sedentary behavior score displays a significant “time × group” interaction effect (*p* = 0.04) with a significant reduction between T0 and T1 in the GT group only (*p* < 0.001). The explicit liking for sedentary activities shows a significant “time × group” interaction (*p* = 0.02) with a significant decrease between T0 and T1 in the GT group only (*p* < 0.001). The explicit wanting for sedentary activities show a significant “time × group” interaction (*p* = 0.02) with a significant decrease between T0 and T1 in the GT group only (*p* < 0.001). The short-term, multi-component, behavioral, educational GT intervention had beneficial effects on primary-school-aged children’s sedentary time and implicit preference for physical over sedentary activities.

## 1. Introduction

The benefits of physical activity (PA) for the physical and mental health and overall well-being of children and adolescents have been clearly described and are now widely accepted by the international scientific community [1,2]. According to the World Health Organization, children and adolescents should accumulate at least 60 min per day of moderate- to vigorous-intensity physical activity (MVPA) (including vigorous-intensity aerobic activities and activities strengthening muscles and bones) at least three days per week [2]. Similarly, screen time should be limited to one hour per day among kids between 2 and 6 years and to two hours per day among kids between 7 and 11 years [2]. Despite a constantly growing global concern for the importance of promoting healthy movement behaviors (physical activity and sedentary behaviors), conclusions from national and international scientific reports continue to show insufficient PA and high levels of sedentary behavior in children and adolescents [3,4,5,6,7,8,9,10,11].

Since children spend most of their waking time at school, the school setting has been suggested as an appropriate and strategic place to develop and implement healthy active living interventions [12]. Interventions aiming at increasing primary school children’s PA level through the implementation of extra exercise/active play sessions have been shown effective [13]. Similarly, more recent studies suggest beneficial effects of interventions that leverage active breaks or active desks to reduce children’s sedentary time [14]. Although such effective interventions should be encouraged and further developed, they can often be expensive, difficult to implement, or unsustainable over time depending on school facilities. Moreover, although these programs have an immediate effect on levels of PA and sedentary behavior, the longer-term effects are less understood [15]. In lieu of prescribed activity interventions, multi-component, health-related behavioral interventions with educational approaches have the potential to induce long-term changes via behavior change strategies [16]. For example, Bernal et al. proposed a multi-component approach among first to fifth grade students over an entire academic year, including intervention focused on the kids (workshops, playground games, or active classroom) combined with actions addressed to parents and teachers (e.g., sensitization, reflective and formative workshops) and changes to the school environment (e.g., physical and material modification of the schoolyard, lunchtime, or recess organization) [12,17]. Children in the intervention group demonstrated an increase in their overall PA level accompanied by a reduction in their sedentary time, with effects remaining stable after one year [12]. Though effective and encouraging, the implementation of such an intervention over a full academic year remains difficult and expensive [12,17].

In that context, the Globe Trotter initiative was developed, proposing an educational multi-component (combining PA challenges, educational sessions, and classical academic lessons incorporating PA and sedentary behavior examples) approach focused on physical activity and sedentary behaviors (movement behaviors) among primary school children over the course of just one month.

The present pilot study evaluates the effect of the Globe Trotter Initiative on PA level, sedentary time, physical fitness, and implicit activity preferences in primary school children.

## 2. Materials and Methods

### 2.1. Population and Design

This study is a cluster-randomized trial piloting the GT initiative. A total of 361 children aged 9 to 10 years old from nine primary schools participated in the study. These nine schools (clusters) were randomly assigned as control schools (CON, without intervention) or Globe Trotter schools (GT). This resulted in four schools (*n* = 121 children) within the CON group and five (*n* = 240 children) within the GT group. At baseline (T0), overall physical fitness, body composition, anthropometric characteristics, PA level, sedentary behaviors, physical self-perception, and implicit activity preferences were evaluated. After the baseline measurements, the children from the GT group followed a one-month educational program incorporating the promotion of healthy movement behaviors into their daily school routine (as described below) while the CON group did not receive any intervention. All measurements were conducted by experts in the field and repeated one month after baseline (T1). In accordance with the Declaration of Helsinki and ethical considerations, all parents and children as well as the teachers and directors of the schools received full informational documents and completed consent forms. This work was conducted in accordance with and under the academic rector and authorities (CPP Sud EST VI).

### 2.2. The Globe Trotter Initiative: From Its Development to Its Implementation

#### 2.2.1. Development of the GT Program

From January 2020, the Judlin De Bouville (JDB) Foundation, as part of its Antéïa Preventive Center, gathered an expert committee in order to develop a school-based educational intervention to promote healthy active living through the use of a collaborative game among primary school children. Public health policymakers (representative of the rectorate and national education cabinet in charge of the primary school policies), preventive education specialists, school nurses, academic and school leaders, and teachers as well as physicians, research scientists, and methodologists were then gathered. Individual interviews and collective meetings gathering specific subgroups to discuss specific questions (e.g., elaboration of the design with researchers and methodologists) were conducted. General meetings gathering the entire expert panel were also conducted. Two main coordinators from the JDB foundation attended all the meetings and conducted all the interviews. From this effort, the GT game was developed, the materials and tools developed and an evaluation process was planned. The entire timetable and material were then presented to the expert panel for final validation.

#### 2.2.2. Implementation of the Program into Schools

First, the schoolteachers completed a one-day training led by experts from the JDB Foundation before the beginning of the game in order to equip them with different tools to be used and how to handle the game. Importantly, the involved teachers, with the help of the investigation and implementation team, proposed content and materials that included the plans, benefits, and disadvantages of PA and sedentary behaviors within their classical academic lessons. Indeed, not just physical education teachers and classes were involved, but all disciplines included in the school curricula were considered (e.g., mathematical or biology lessons included PA and sedentary behavior examples). A member of the JDB foundation remained available to help teachers at any time during the game.

#### 2.2.3. The GT Intervention

Briefly, this GT game aimed to promote daily PA among children over a one-month period, encouraging the class group to reach the symbolic equivalent of 38,000 km, representing an entire tour around the globe. Every single movement counted and every 15 min of movement corresponded to approximately one km. Each child, along with their entire class, attended four educational classes over the course of the month dedicated to the promotion and consciousness of healthy active living. Each child had a “travel book” in which they indicated objectives and how they would encourage friends and family to get involved and active. The activity time of each child and their entourage was then converted in km and cumulated, making GT a collaborative game.

### 2.3. Measurements

#### 2.3.1. Anthropometric Measurements

Weight (kg) and height (cm) were measured by a trained experimenter using a standard apparatus (using a weighting scale and standardized wall-mounted stadiometer Seca, Lea Mureaux) to the nearest 0.1 kg and 0.5 cm, respectively. Body mass index (BMI) was calculated as body weight divided by height squared (and expressed in kg/m^2^). Children were classified by weight status, including obesity (95th to 99th percentile) and severe obesity (≥99th percentile), according to BMI curves, chronological age, and sex (Centers for Disease Control and Prevention—CDC) [18].

#### 2.3.2. Physical Fitness Tests

##### Muscular Strength

Upper body muscular strength was measured with a handgrip dynamometer (Takei Scientific Instruments Co., Ltd.). A previous study reported acceptable inter-trial reliability for the hand dynamometer [19,20]. Participants were asked to adjust the handgrip bar so that the second joint of the fingers was bent to grip the handle of the dynamometer. They then stood upright with their arm in a vertical position and the dynamometer close to the body and were asked to squeeze the hand dynamometer as hard as possible. The test was completed twice on the dominant arm and the best performance was retained. Participants also performed a medicine ball throw (MBT) test that measured the explosive power of the upper limbs. Participants were seated in a chair with their feet shoulder-width apart and their backs supported so that only upper body strength was used. The medicine ball was held at shoulder height and thrown forward with a counter-movement of the forearms. They were asked to throw the medicine ball as far as possible. The test was performed twice and the research staff recorded the distance between the front chair leg and the medicine ball landing site (to the nearest 0.5 cm). The MBT is validated and reliable in children and adolescents with an intra-class coefficient = 0.98 [20]. Finally, lower limb muscle strength was assessed during a counter movement jump (CMJ) using Optojump technology (Microgate SRL, Rome, Italy) [21].

##### Flexibility

The sit and reach test is a common measure of flexibility, and measures the flexibility of the lower back and hamstring muscles [22]. For this test, participants were asked to sit on the floor with legs stretched out straight ahead. Shoes should be removed. The soles of the feet were placed flat against the box. Both knees should be locked and pressed flat to the floor—the tester may assist by holding them down. With the palms facing downwards, and the hands on top of each other or side by side, the subject reaches forward along the measuring line as far as possible. After two practice reaches, the subject reaches out and holds that position for at least one to two seconds while the distance is recorded. This test was performed twice and the best values were retained. Research staff ensured there were no jerky movements during the measurement trial [23].

##### Coordination/Motor Skills

Coordination and motor skills were assessed using an obstacle course that was developed to facilitate field setting group testing among children [24]. An obstacle course assessment is a dynamic setting incorporating both object control and locomotor skills while the child moves through a timed and scored obstacle course. The time needed to complete the obstacle course was measured using a classical stopwatch. Participants completed the obstacle course twice, the first time being a familiarization try. This obstacle course was developed from the previously detailed work of Larouche et al. (2014) [24].

#### 2.3.3. Physical Activity Level and Sedentary Behaviors

PA level and sedentary behaviors were assessed using the Children and Adolescents Physical Activity and Sedentary Behaviors Questionnaire (CAPAS-Q). The CAPAS-Q is a self-administered questionnaire containing 31 items developed to assess 7-day PA and sedentary behavior during a typical week. This questionnaire assesses PA time, duration, and intensity as well as its context of practice (school and non-school, sports and leisure). Similarly, it evaluates the time dedicated to sedentary behaviors and discriminates between screen or non-screen behaviors, as well as their consecutive duration. The context of sedentary behaviors is also assessed (school, non-school, or transportation setting). The reproducibility, validity, and reliability of this questionnaire in French children and adolescents (and in French language) has been previously reported [25].

#### 2.3.4. Physical Self-Perception

Physical self-perception was measured with the French version of the short form of the Physical Self-Description Questionnaire [26,27]. This questionnaire contains 40 items which measure physical self-dimensions: coordination, strength, flexibility, endurance, global self-esteem, health, activity, body fat, sport competence, global physical self-concept, and appearance. A 6-point scale from 1 (false) to 6 (true) was used to assess each item. The eleven individual scores were computed to obtain a global score for physical self-perception. The validity of this questionnaire in children and adolescents has been previously reported [28].

#### 2.3.5. Implicit Activity Preferences

The Activity Preference Assessment (APA) [29] is a computerized behavioral task designed to assess and quantify biases in decision-making across multiple leisure time activities [29]. The APA is administered on a desktop computer via E-Prime (Psychology Software Tools, Sharpsburg, PA, USA) and takes approximately 10 min to complete. Each participant was first asked how much they like to do and want to do a variety of common physical activities (e.g., ball sports, swimming) and sedentary activities (e.g., arts and crafts, watching TV) on visual analog scales to quantify explicit liking and explicit wanting (range 0 to 100). They then completed a forced-choice paradigm, or “would you rather?” game. Out of each pair of activity images (4 sets of 30 pairs, with breaks between sets), they were asked to select as quickly as possible the activity they most wanted to do to assess implicit wanting. Of the 120 pairs, 64 were sedentary versus physical activities, with the remaining pairs falling within-category. Every pair was unique and all possible comparisons were made. Implicit wanting scores from the head-to-head comparisons of sedentary versus physical activities took into account choices made and reaction times. These scores were used to compute the bias score (range −100 to +100), quantifying the relative implicit preference. Positive scores represent a relative preference towards sedentary activities and negative scores represent a relative preference towards physical activities. Data was processed via an automated scoring procedure in Anaconda3 (Austin, TX, USA). The APA was originally developed and validated in English [29], but the data of the French version are presented here. All text within the APA and the corresponding participant instructions were translated to French by a bilingual French and English speaker, then back-translated to English to confirm that the original meaning of all text was retained.

### 2.4. Statistical Analyses

Sample size was estimated according to (i) the CONSORT 2010 statement with extension to randomized pilot trials and (ii) Cohen’s recommendations, which define effect-size bounds as small (ES: 0.2), medium (ES: 0.5) and large (ES: 0.8, “grossly perceptible and therefore large”). With 100 participants (33 and 66 children, unbalanced in favor to GT group), an effect size greater than 0.6 could be obtained for a two-sided type I error at 0.05 and 80% statistical power. As this study was a cluster-randomized trial, the sample size needed to be inflated to take into account between- and within-school variability. For an intra-class correlation coefficient at 0.05 and considering around 25 children by school, at least 218 children in 9 classes were necessary.

Statistical analyses were performed using Stata software (version 15; StataCorp, College Station, TX, USA). All tests were two-sided, with an alpha level set at 5%. Categorical data were expressed as number of children and associated percentages, and continuous data as mean ± standard deviation.

Longitudinal analyses were performed using linear mixed models for quantitative dependent variables (e.g., grip strength) and generalized linear mixed models with logit link functions for binary dependent variables (e.g., sedentary bias). In these models, the following fixed effects were studied: baseline value of the studied variable, group (CON and GT), time of measurement (T0 and T1) and their interaction (time × group). The schools and children were considered random effects. Sensitivity analyses were performed in order to evaluate the impact of missing data on the results, considering for each variable only children with complete data (at both T0 and T1).

In the GT group, Spearman’s correlation coefficients (rho) were calculated between baseline levels of physical activity and sedentary behaviors and the change between T0 and T1 in each of the parameters was studied. The changes were calculated in percentage, except for flexibility (because of zero values), for which a simple difference was calculated (division by zero is not possible). Spearman’s correlation coefficients were then interpreted as follows (absolute value): ≥0.70 (strong correlation), 0.50–0.69 (moderate correlation), 0.30–0.49 (low correlation), 0.00–0.29 (no or negligible correlation).

## 3. Results

### 3.1. Study Population

Of the initial 361 children enrolled, 339 completed both evaluation times (T0 and T1) (116 in the CON group and 223 in the GT group), 8 only completed T0 evaluation (2 in the CON group and 6 in the GT group), 9 only completed T1 evaluation (2 in the CON group and 7 in the GT group), and 5 did not complete either the T0 or T1 evaluations (1 in the CON group and 4 in the GT group). The present analysis was performed on the 347 children (43.2% boys, 41.5% in the CON group and 44.1% in the GT group) who at least completed T0 evaluation.

The baseline mean body weight for the whole sample was 35.0 ± 7.6 kg (*n* = 279), with 35.0 ± 7.1 kg (*n* = 85) and 35.0 ± 7.9 kg (*n* = 194) for the CON and GT groups (*p* = 0.99), respectively. The baseline BMI for the whole sample was 17.5 ± 2.8 kg/m^2^ (*n* = 279), with 17.7 ± 2.5 kg/m^2^ (*n* = 85) and 17.3 ± 3.0 kg/m^2^ (*n* = 194) for the CON and GT groups (*p* = 0.23), respectively.

### 3.2. Intervention Effects

There were no significant differences between group or time for the CMJ height and duration, the MBT distance, and flexibility. Both the grip strength performance and overall completion time of the obstacle course show a significant time effect (*p* < 0.001) in both groups, without significant group effect (Table 1).

The sedentary behavior score displays a significant “time × group” interaction effect (*p* = 0.04), with a significant reduction in sedentary behavior between T0 and T1 in the GT group only (*p* < 0.001). The PA level score and physical self-perception score do not show any significant time nor group effects (Table 1).

The explicit liking for sedentary activities scores shows a significant “time × group” interaction (*p* = 0.02 for main analysis and *p* = 0.03 for sensitivity analysis) with a significant decrease between T0 and T1 in the GT group only (*p* < 0.001). Similarly, the explicit wanting for sedentary activities show a significant “time × group” interaction (*p* = 0.02 for both main and sensitivity analyses) with a significant decrease between T0 and T1 in the GT group only (*p* < 0.001). The overall bias score for implicit activity preferences shows a significant “time × group” interaction (*p* = 0.002 for main analysis and *p* = 0.004 for sensitivity analysis) with a significant decrease between T0 and T1 in the GT group (*p* = 0.002 for main analysis and *p* = 0.003 for sensitivity analysis), suggesting a shift towards a PA preference (Table 2).

The dichotomous categorization for bias scores shows a significant “time × group” interaction (*p* = 0.005 for main analysis and *p* = 0.01 for sensitivity analysis) with a significant decrease between T0 and T1 in the number of children who prefer sedentary activities among the GT group only (*p* = 0.004 for main analysis and *p* = 0.005 for sensitivity analysis) compared to an increase in the CON group.

### 3.3. Effect of the Intervention by Baseline Activity Levels

Table 3 presents the correlations between the baseline levels of the PA and sedentary behaviors of the children who received the intervention (GT group) and change in each of the parameters studied between T0 and T1. There was a negative correlation between the baseline level of PA and change in PA levels from T0 to T1 (rho = −0.445, *p* < 0.001), as well as sedentary behavior level (rho = −0.155, *p* = 0.02). Similarly, there was a negative correlation between the baseline level of sedentary behaviors and change in sedentary behavior level between T0 and T1 (rho = −0.377, *p* < 0.001).

## 4. Discussion

The present pilot work evaluates the effects of a novel, one-month educational intervention (the Globe Trotter Initiative), aiming to simultaneously engage primary school children in an activity-based game while increasing knowledge of the health effects of PA and sedentary behavior though targeted educational sessions as well as classical academic lessons integrating healthy behaviors as teaching materials.

The results of the present analysis show that the Globe Trotter Initiative that combined educational sessions, a gaming context, and the use of topic-specific material as academic teaching support seem effective in reducing primary school children’s sedentary time as well as increasing their preferences for physical activities over sedentary behaviors.

The present results seem particularly promising, indicating that a one-month school-based intervention might be able to reduce sedentary time in primary-school-aged children. Importantly, these results were reinforced in the present work by a reduction in children’s implicit preference for sedentary activities, with an increasing preference for PA over time.

Our results are the first to provide such evidence regarding the ability to change the implicit preference for movement behaviors, based on a validated and reliable computerized behavioral task [29]. Indeed, while school-based interventions implementing extra PA sessions [30,31,32], active recesses [33,34], or active breaks, classes, and desks [35,36,37] have been shown effective in increasing kids’ PA level and overall health, few to our knowledge have proposed such a combined approach targeting movement behaviors. In previous work, Harrison and colleagues conducted a controlled 16-week intervention among primary school kids based on health education sessions and professional support to teachers [38]. The intervention consisted of ten 30-min lessons aiming to individually promoting PA and reduce sedentary behavior. The results of this previous trial show that while self-reported PA time and self-efficacy related to PA were improved in the intervention schools, sedentary time remained unchanged [38]. Their results are in line with the literature describing great difficulty in reducing sedentary time among children, despite the ability to improve the level of MVPA [39,40]. Although more difficult to induce, behavioral improvements in terms of sedentary time have been recently suggested as necessary to improve youths’ overall health independently of their level of PA [41,42,43,44,45]. Indeed, cardio-metabolic health [44], hepatic profile [46], appetite control [43,47,48], and overall health [43] of children and adolescents may be primarily determined by sedentary time, regardless of PA level. The GT initiative shows, then, highly promising results by significantly improving the sedentary time of the kids while reducing their preference for sedentary behaviors.

Interestingly, our results also indicate that those with higher baseline levels of sedentary behavior were associated with greater changes in sedentary time over the intervention, suggesting a greater effect on the kids initially presenting a high time dedicated to sedentary behaviors, which is encouraging. This highlights once more the need to consider children’s initial behavioral profile to propose more individualized and effective interventions [41]. Although our results did not show any intervention-specific improvement in the children’s physical fitness, nor any effect of the children’s initial physical fitness profile, such results were expected since the Globe Trotter program rests solely on an educational approach for just one month and does not consist in any physical training. Meaningful changes in physical fitness likely take more time to manifest and longer-term follow-up is needed.

The present results must be interpreted in light of some limitations. Despite the strength of the sample size and the use of objective field testing to evaluate the children’s physical fitness, the use of a self-reported questionnaire to assess PA and sedentary behaviors may be considered a limitation. However, the CAPAS-Q was recently found reliable and reproducible in this population, validated against objective accelerometer-based measurements [25]. Moreover, the results obtained using the questionnaire (reduced sedentary time) are reinforced by the results obtained using the APA [29]. The absence of follow-up is certainly the main limitation of the present work and further evaluations of the Globe Trotter program are needed to assess its long-term impact. Similarly, the absence of control of the completion rate in the intervention group comprises a limitation of the present work.

## 5. Conclusions

A short-term, multi-component, behavioral, educational intervention targeting movement behaviors called the Globe Trotter Initiative, while unable to impact physical activity level and overall fitness, led to beneficial effects on primary-school-aged children’s sedentary time and implicit preference for physical over sedentary activities. Further studies evaluating the longer effects of the Globe Trotter program and similar interventions are needed. Similarly, future studies should better consider the baseline physical and behavioral profile of the children in order to developed individualized intervention targeting the specific needs of the children.

## Figures and Tables

**Table 1:** Physical fitness, physical activity level, sedentary time, and perceived physical fitness at baseline (T0) and one month later (T1) according to control (CON) and Globe Trotter (GT) groups.

	Whole Sample		CON		GT				
	*n*	Mean ± SD	*n*	Mean ± SD	*n*	Mean ± SD	p^g^	p^t^	p^i^
CMJ height (cm)									
T0	225	20.4 ± 4.3	67	20.2 ± 4.2	158	20.5 ± 4.4	0.91	0.36	0.28
T1	257	20.6 ± 4.4	65	19.8 ± 4.5	192	20.8 ± 4.3			
T0	217	20.3 ± 4.3	64	20.0 ± 4.1	153	20.4 ± 4.3	0.96	0.41	0.31
T1	217	20.3 ± 4.4	64	19.7 ± 4.5	153	20.6 ± 4.3			
CMJ duration (s)									
T0	225	0.41 ± 0.04	67	0.40 ± 0.04	158	0.41 ± 0.05	0.95	0.33	0.22
T1	257	0.41 ± 0.04	65	0.40 ± 0.05	192	0.41 ± 0.04			
T0	217	0.40 ± 0.04	64	0.40 ± 0.04	153	0.41 ± 0.04	0.99	0.38	0.25
T1	217	0.40 ± 0.05	64	0.40 ± 0.05	153	0.41 ± 0.04			
MBT distance (cm)									
T0	261	240 ± 49	67	231 ± 47	194	243 ± 50	0.67	0.57	0.89
T1	282	243 ± 45	64	233 ± 42	218	246 ± 45			
T0	252	239 ± 49	63	230 ± 45	189	242 ± 50	0.65	0.55	0.92
T1	252	241 ± 44	63	232 ± 41	189	245 ± 45			
Grip strength (N)									
T0	283	14.5 ± 3.7	88	14.1 ± 3.2	195	14.7 ± 3.8	0.93	<0.001	0.43
T1	303	15.6 ± 3.8	85	15.1 ± 3.3 ***	218	15.7 ± 4.0 ***			
T0	273	14.4 ± 3.6	83	13.9 ± 3.1	190	14.5 ± 3.8	0.92	<0.001	0.43
T1	273	15.4 ± 3.8	83	15.2 ± 3.3 ***	190	15.6 ± 4.0 ***			
Flexibility (cm)									
T0	282	0.70 ± 7.45	88	1.04 ± 6.89	194	0.55 ± 7.71	0.99	0.11	0.12
T1	304	0.95 ± 7.62	86	1.49 ± 7.00	218	0.73 ± 7.86			
T0	273	0.79 ± 7.39	84	1.06 ± 6.79	189	0.68 ± 7.66	0.99	0.12	0.13
T1	273	0.90 ± 7.75	84	1.62 ± 7.00	189	0.58 ± 8.06			
Obstacle course time (s)									
T0	281	26.5 ± 4.3	88	26.5 ± 3.9	193	26.6 ± 4.4	0.96	<0.001	0.52
T1	302	24.6 ± 4.2	85	24.8 ± 4.0 ***	217	24.5 ± 4.3 ***			
T0	270	26.6 ± 4.3	82	26.4 ± 3.9	188	26.6 ± 4.4	0.98	<0.001	0.48
T1	270	24.7 ± 4.3	82	24.8 ± 4.0 ***	188	24.7 ± 4.4 ***			
Sedentary score									
T0	342	2.06 ± 0.60	117	2.09 ± 0.63	225	2.05 ± 0.58	0.69	0.36	0.04
T1	328	1.94 ± 0.67	115	2.03 ± 0.73	213	1.89 ± 0.63 ***			
T0	328	2.07 ± 0.60	115	2.08 ± 0.62	213	2.07 ± 0.58	0.74	0.39	0.04
T1	328	1.94 ± 0.67	115	2.03 ± 0.73	213	1.89 ± 0.63 ***			
Physical activity score									
T0	344	2.49 ± 0.52	118	2.35 ± 0.56	226	2.56 ± 0.49	0.30	0.98	0.39
T1	331	2.53 ± 0.57	114	2.37 ± 0.62	217	2.61 ± 0.52			
T0	330	2.50 ± 0.52	114	2.37 ± 0.55	216	2.57 ± 0.50	0.33	0.97	0.38
T1	330	2.53 ± 0.57	114	2.37 ± 0.62	216	2.62 ± 0.52			
Physical self-perception score									
T0	340	4.59 ± 0.75	117	4.58 ± 0.70	223	4.59 ± 0.77	0.80	0.62	0.12
T1	327	4.62 ± 0.78	115	4.55 ± 0.77	212	4.65 ± 0.78 *			
T0	324	4.58 ± 0.75	114	4.58 ± 0.71	210	4.59 ± 0.77	0.79	0.63	0.13
T1	324	4.61 ± 0.78	114	4.56 ± 0.77	210	4.65 ± 0.78 *			

CMJ: counter movement jump; CON: control group; GT: Globe Trotter group; MBT: medicine ball throw; *n*: number of children; p^g^: p-value of group effect; p^i^: *p*-value of interaction (time × group) effect; p^t^: *p*-value of time effect; SD: standard deviation; T0: baseline; T1: one month after baseline. The stars represent the results of the subgroup analyzed: *: *p* < 0.05, ***: *p* < 0.001 compared to T0. All analyses were carried out using linear mixed models considering an adjustment for the baseline value of the studied variable. For each variable, two analyses were performed: the first one on all the data available, and the second one only on the sample of participants with complete data (at both T0 and T1).

**Table 2:** Results from the cognitive activity preference assessment (APA) at baseline (T0) and one month later (T1) according to control (CON) and Globe Trotter (GT) groups.

	Whole Sample		CON		GT				
	*n*	Mean ± SD or *n* (%)	*n*	Mean ± SD or *n* (%)	*n*	Mean ± SD or *n* (%)	p^g^	p^t^	p^i^
Explicitly liking SED									
T0	275	69.1 ± 13.7	104	70.3 ± 13.2	171	68.3 ± 14.0	0.77	0.36	0.02
T1	239	66.8 ± 14.7	88	70.3 ± 13.4	151	64.8 ± 15.1 ***			
T0	219	69.9 ± 13.4	79	70.7 ± 13.0	140	69.4 ± 13.6	0.85	0.39	0.03
T1	219	66.3 ± 15.0	79	69.5 ± 13.9	140	64.4 ± 15.4 ***			
Explicitly liking PA									
T0	275	65.6 ± 14.6	104	62.8 ± 16.0	171	67.3 ± 13.4	0.38	0.40	0.18
T1	239	65.7 ± 13.7	88	64.4 ± 14.4	151	66.4 ± 13.3			
T0	219	65.6 ± 13.9	79	62.4 ± 15.0	140	67.5 ± 12.9	0.38	0.42	0.24
T1	219	65.4 ± 13.8	79	63.4 ± 14.0	140	66.6 ± 13.6			
Explicitly wanting SED									
T0	275	63.3 ± 14.6	104	64.7 ± 14.3	171	62.5 ± 14.7	0.70	0.31	0.02
T1	239	60.1 ± 15.7	88	63.8 ± 14.9	151	57.9 ± 15.8 ***			
T0	219	63.8 ± 14.5	79	64.5 ± 15.0	140	63.4 ± 14.3	0.85	0.38	0.02
T1	219	59.4 ± 15.8	79	63.1 ± 15.2	140	57.3 ± 15.8 ***			
Explicitly wanting PA									
T0	275	59.3 ± 15.3	104	57.9 ± 14.7	171	60.2 ± 15.6	0.70	0.004	0.06
T1	239	57.8 ± 15.9	88	55.2 ± 16.5 **	151	59.4 ± 15.4			
T0	219	58.8 ± 15.5	79	57.5 ± 14.8	140	59.5 ± 15.9	0.68	0.02	0.08
T1	219	57.3 ± 15.9	79	53.8 ± 15.5 *	140	59.2 ± 15.8			
APA bias									
T0	274	−8.4 ± 60.5	104	4.9 ± 58.2	170	−16.5 ± 60.6	0.35	0.15	0.002
T1	239	−13.0 ± 56.7	88	8.1 ± 54.0	151	−25.3 ± 54.7 **			
T0	219	−7.5 ± 55.9	79	2.3 ± 59.6	140	−13.0 ± 53.2	0.44	0.19	0.004
T1	219	−12.7 ± 56.5	79	9.2 ± 53.8	140	−25.1 ± 54.3 **			
SED bias									
T0	274	126 (46.0)	104	52 (50.0)	170	74 (43.5)	0.67	0.20	0.005
T1	239	97 (40.6)	88	48 (54.5)	151	49 (32.5) **			
T0	219	103 (47.0)	79	41 (51.9)	140	62 (44.3)	0.57	0.38	0.01
T1	219	90 (41.1)	79	45 (57.0)	140	45 (32.1) **			

APA: activity preference assessment; *n*: number of children; PA: physical activity; p^g^: *p*-value of group effect; p^i^: *p*-value of interaction (tim × group) effect; p^t^: *p*-value of time effect; SD: standard deviation; SED: sedentary; T0: baseline; T1: one month after baseline. The stars represent the results of the subgroup analyzed: *: *p* < 0.05, **: *p* < 0.01, ***: *p* < 0.001 compared to T0. All analyses were carried out using linear mixed models considering an adjustment for the baseline value of the studied variable. For each variable, two analyses were performed: the first one on all the data available, and the second one only on the sample of participants with complete data (at both T0 and T1).

**Table 3:** Correlations between the baseline levels of the physical activity and sedentary behaviors of the children (Globe Trotter group only), and change in each of the parameters studied between baseline (T0) and one month later (T1).

	Physical Activity Level at T0			Sedentary Behavior Level at T0		
	*n*	rho	*p*	*n*	rho	*p*
Change in CMJ height	152	−0.105	0.20	153	−0.147	0.07
Change in CMJ duration	152	−0.092	0.26	153	−0.159	0.049
Change in MBT distance	188	−0.059	0.42	189	0.008	0.91
Change in grip strength	189	−0.011	0.88	190	0.046	0.53
Change in flexibility *	188	0.004	0.96	189	0.143	0.049
Change in obstacle course time	187	0.016	0.83	188	0.014	0.85
Change in sedentary score	212	−0.155	0.02	213	−0.377	<0.001
Change in physical activity score	216	−0.445	<0.001	216	0.075	0.27
Change in physical self-perception score	209	−0.004	0.95	210	−0.010	0.89
Change in explicitly liking SED	140	−0.060	0.48	140	0.095	0.27
Change in explicitly liking PA	140	0.041	0.63	140	−0.111	0.19
Change in explicitly wanting SED	140	−0.089	0.30	140	−0.002	0.99
Change in explicitly wanting PA	140	−0.108	0.21	140	−0.208	0.01
Change in APA bias score	140	−0.077	0.37	140	0.054	0.53

APA: activity preference assessment; CMJ: counter movement jump; *n*: number of children; PA: physical activity; rho: Spearman’s correlation coefficient; SED: sedentary; T0: baseline; T1: one month after baseline. Spearman’s correlation coefficients were interpreted as follows (absolute value): ≥0.70 (strong correlation), 0.50–0.69 (moderate correlation), 0.30–0.49 (low correlation), 0.00–0.29 (no or negligible correlation). *: Because zero values were possible for flexibility, percentage change could not be calculated and a simple difference between T0 and T1 values was calculated.

## Data Availability

Due to reglementary conditions, data are not avaiblable.
